# Determination of Thyroid Hormones and 11 Metabolites in the Human Serum Using a Simple Derivatization Strategy and Analysis by Isotope-Dilution Liquid Chromatography Tandem Mass Spectrometry

**DOI:** 10.1021/acs.analchem.5c00714

**Published:** 2025-04-23

**Authors:** Jiří Kohoutek, Juan I. Sánchez-Avila, Marie Smutná, Petr Janků, Jana Klánová, Klára Hilscherová

**Affiliations:** † RECETOX, Faculty of Science, Masaryk University, Kotlarska 2, 602 00 Brno, Czech Republic; ‡ CeMM Research Center for Molecular Medicine of the Austrian Academy of Sciences, Lazarettgasse 14, AKH BT 25.3, 1090 Vienna, Austria; § Clinic of Gynecology and Obstetrics, University Hospital Brno, Jihlavska 20, 625 00 Brno, Czech Republic; ∥ Department of Health Sciences, Faculty of Medicine, Masaryk University, Kamenice 126/3, 625 00 Brno, Czech Republic

## Abstract

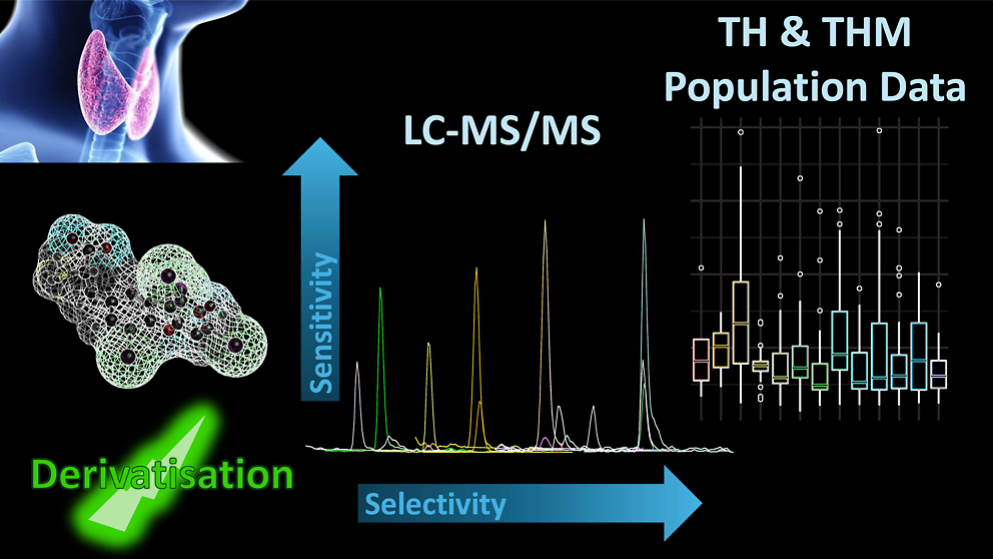

Many analytical methods for thyroid hormone (TH) determination lack sensitivity and/or specificity. The thyroid hormone metabolites (THMs) are usually not measured at all. This study describes the development of sensitive high-throughput analytical methods for determining the total concentration and free fraction of TH and THM in the human serum. For the analysis of the TOTAL fraction, we employed protein precipitation and anionic exchanger solid-phase extraction. For the FREE fraction, ultrafiltration and salt-out liquid partitioning were used. Derivatization using dansyl chloride was employed to enhance the sensitivity of HPLC-ESI-MS/MS analysis. Both protocols were validated according to the European Analytical Guidelines (2002/657/EC). We obtained very good recoveries (73–115%) and precision. Interday coefficients of variation (CVs) for most of the analytes ranged from 1.2 to 16.4%. The sensitivity was excellent with detection limits in the sub ppt range for the majority of TH and THM. A significant enhancement in sensitivity (>10 fold) was achieved through derivatization. The applicability was proved on a set of samples from pregnant women enrolled in the CELSPAC cohort (*n* = 120). Our TH reference ranges are in good agreement with those reported in the literature. The methods also allowed us to quantify the levels of 11 THM, including some previously undetected THM in total and free fractions, and proved to be suitable for high-throughput routine TH and THM analyses. Our approach offers an important advancement in thyroid hormone analysis. To the best of our knowledge, it is for the first time that data for T1A and T2A as well as for free THM levels in the human serum are published in the literature. Moreover, our study also brings the first information about the levels of most of the THM in pregnant women.

## Introduction

Thyroid hormones (THs) are important endogenous iodinated amino-acid-derived biomolecules with signaling properties.^1^ TH production and secretion is regulated via the hypothalamus–pituitary–thyroid axis. Serum TH levels are tightly regulated under physiological conditions.^2^ TH includes prohormone l-thyroxine (3,3′,5,5′-tetraiodo-l-thyronine; T4) and 3,3′,5-triiodothyronine (T3). The majority of T3 is formed by the deiodination of T4.^3^ T4 can also be deiodinated to form an inactive metabolite of T3, the 3,3′,5′-triiodothyronine (rT3).^1^ Similarly, deiodination of T3 generates either the active 3,5-diiodothyronine (3,5-T2) or the 3,3′-diiodothyronine (3,3′-T2).^4,5^ Besides deiodination, several other pathways of TH metabolism are also possible. TH metabolites (THM), include thyronamines (TAms), resulting from TH decarboxylation, and thyroacetic acids (TAc) resulting from the deamination of TAm. Sulfation and glucuronidation are additional pathways in the metabolism of TH.^2^ Circulating the T4 concentration is 50–60 times higher compared to T3, and both hormones are also partially bound to blood proteins.^4,6,7^ As a result, only a very small unbound free fraction of these hormones (0.01% of total T4 and 0.2% of total T3 as determined by LC–MS/MS;^8^) remains available to directly access the target organs.^4,6,7,9^ For many THM, their function is still unclear. The full names, abbreviations, as well as molecular structures of analyzed thyroid hormones and their metabolites and introductory information on their biological activity or effects are provided in Supporting Information (Table S1). For the analysis of total and free thyroid hormones, the widely used methods have been (radio)immunoassays (RIA/IA).^7,10,11^ Despite high sensitivity, immunoassays often lack adequate specificity.^7,10,11^ Over the years, the progress in mass spectrometry-based techniques (namely, LC–MS/MS) has led to increasing replacement of RIAs in TH analysis.^7,10−13^ The earliest LC–MS methods were developed for human and animal serum/plasma only for the analysis of T4 and T3.^12,14,15^ Later published methods included the analysis of rT3,^3,7^ 3,3′-T2,^16^ and 3,5-T2^13^ metabolites and, more recently, T3A and T4A.^17^ However, there are still obstacles limiting the full use of LC–MS/MS potential, particularly regarding sensitivity in different modes and the ionization efficiency of TH and THM.^10,17^

Derivatization is an alternative approach used in sample preparation for LC–MS/MS. It is often used to separate the masses of the derivatized products from the interfering compounds^18,19^ and/or to improve ionization efficiency and thus signal intensity.^18,20^ Because TH and some THM can be detected by LC–MS/MS without labeling, derivatization was rarely tested. All TH and THM contain functional groups that can be derivatized, specifically a phenolic hydroxyl group and an amino group. Derivatization approaches, such as dansylation, have been used to label phenolic hydroxyl and/or amine groups.^18,21^ Despite its benefits, derivatization is rarely used in the fields of TH and THM analyses^22,23^ and its potential in combination with mass spectrometry detection has not been fully exploited.

The process of sample preparation presents another unique set of challenges. The stability of analytes, careful selection of additives and reagents, and compatibility with the derivatization step must be considered. In addition, a purification and preconcentration method must be chosen to match physicochemical properties and low abundance of most analytes and also the limited sample volume.^7,17,24,25^ In the case of free fraction, a data bias due to the equilibrium shifts should be avoided.^24,26,27^

In this study, we describe the development and application of methods to analyze the total concentration and free fraction of TH (T3, T4) and their 11 metabolites (T0, 3-T1, 3′-T1, 3,3′-T2, 3,5-T2, rT3, T1Am, T1A, T2A, T3A, and T4A; full names in Table 1) in the human serum. For the analysis of the TOTAL fraction, we employed protein precipitation and solid phase extraction (SPE) with anionic-exchange cartridges. For the FREE fraction, ultrafiltration and salt-out liquid partitioning were used. The next step includes derivatization using dansyl chloride and final analyses by HPLC-ESI-MS/MS. We assessed the validation parameters of the methods, including sensitivity, reliability, and robustness. Their applicability was demonstrated on a set of real serum samples from the CELSPAC:TNG cohort.

**Table 1:** Linearity and Method Limits of Quantification (MQL) for TH and THM

		TOTAL fraction		FREE fraction	
	analyte	linear range (pg/mL)	MQL (pg/mL)	Linear range (pg/mL)	MQL (pg/mL)
T0	thyronine	1–160	0.9	1.6–160	1.6
3-T1	3-iodothyronine	10–160	6.5	0.3–160	0.3
3′-T1	3′-iodothyronine	0.2–120	0.2	0.4–120	0.2
3,3′-T2	3,3′-diiodothyronine	1–160	0.4	0.3–160	0.2
3,5-T2	3,5 -diiodothyronine	1.3–160	1.2	0.1–160	0.1
T3	3,3′,5 -triiodothyronine	1.1−3200	1.1	0.9–3200	0.8
rT3	3,3′,5′-triiodothyronine	1.9–1600	1.9	0.4–1600	0.4
T4	3,3′,5,5′-tetraiodo-l-thyronine (l-thyroxine)	71–64,000	70.6	2.3–64,000	2.3
T1Am	3-iodothyronamine	6–130	4.6	1.0–130	0.5
T1A	3-iodothyroacetic acid	1–160	0.7	0.3–160	0.3
T2A	3,5-diiodothyroacetic acid	2–160	0.6	0.1–160	0.1
T3A	3,3′,5-triiodothyroacetic acid (Triac)	5–130	3.3	0.3–130	0.3
T4A	3,3′,5,5′-tetraiodothyroacetic acid (Tetrac)	2–160	1.8	0.2–160	0.2

## Experimental Section

### Chemicals and Reagents

Individual standards (purity >99%) of T4, T3, rT3, 3,3′-T2, 3,5-T2, T1Am, and T3A and individual solutions of internal standards ^13^C_6_-T4, ^13^C_6_-T3, ^13^C_6_-rT3, and ^13^C_6_-3,3′-T2 were purchased from Sigma (St. Louis, MO, USA). Individual standards (purity >95%) of T0, 3-T1, 3′-T1, T1A, T2A, T4A, and the internal standard ^13^C_6_-T1Am were purchased from Toronto Research Chemicals (ON, Canada). HPLC grade solvents, acetone (ACE), acetonitrile (MeCN), and methanol (MeOH), and the reagents, formic acid (FA), citric acid, ammonium hydroxide (NH_4_OH, 25% purity), and dansyl chloride (dnsCl, 99% purity), were purchased from Sigma-Aldrich. Ultrapure grade water (18.2 MΩ·cm, total organic carbon ≤ 2 ng/mL) was obtained from an ultrafiltration device with a UV lamp (Direct-Q 3UV, Merck Millipore, Darmstadt, Germany). The T3/T4 depleted human serum (total T4 ≤ 20 nmol/L (≤15.5 ng/mL), total T3 ≤ 1 nmol/L (≤0.65 ng/mL), free T4 ≤ 1 pmol/L (≤0.78 pg/mL), and free T3 ≤ 5 pmol/L (≤3.25 pg/mL)) was purchased from BBI Solutions (Crumlin, UK) and used in all optimalization and recovery test experiments.

### Serum Sample Preparation

The general strategy of sample preparation for the analysis of the total and free fraction was based on the methods published by Jongejan et al.^17^ and Tanoue et al.^24^ However, we optimized both protocols to fulfill our performance criteria. Attention was paid especially to the selection of solvents, antioxidants, SPE sorbents, ultrafiltration devices, and the overall compatibility with the derivatization step. Due to the possible light sensitivity of TH and THM, we tried to avoid light and heat as well as unnecessary idle time during sample preparation. Amber glass and/or aluminum foil coverage were used whenever possible.

### TOTAL Fraction

200 μL of homogenized serum sample was transferred into a 1.5 mL Eppendorf tube and spiked with 20 μL of the ISTD solution (^13^C_6_-T4 400 ng/mL, ^13^C_6_-T3 10 ng/mL, ^13^C_6_-rT3 6 ng/mL, ^13^C_6_-3,3′-T2 2 ng/mL, and ^13^C_6_-T1Am 2 ng/mL) in water and 70 μL of the antioxidant (25 mg/mL citric acid in water). The mixture was vortexed for 1 min and equilibrated for 45 min at room temperature. Then, 750 μL of ice-cold ACE was added. The samples were vortexed (20s) and sonicated for 5 min (crushed ice cooled bath). Subsequently, the samples were centrifuged at 2500*g* and 4 °C for 10 min. The supernatant was transferred to a clean 96-well plate and the solvent was evaporated at 50 °C under nitrogen (30 psi, approx.35 min). 1.5 mL of 5% NH_4_OH and 5% MeOH in water was added to the dried samples, and the mixture was sonicated for 1 min. The resuspended samples were subjected to SPE extraction using an Oasis MAX 96-well Plate (60 mg Sorbent/well, 30 μm), which had been previously activated with 2 mL of 5% NH_4_OH in MeOH and equilibrated with 2 mL of 5% NH_4_OH in water. Interferences were washed out with 2 mL of 5% NH_4_OH in water and 2 mL of MeOH. Then, the well plate was dried for 2 min with air. TH and THM were eluted with 1.5 mL of 3% formic acid in acetone. Eluates were transferred to clean amber conical glass vials and evaporated to dryness using a slow flow of nitrogen at 50 °C.

### FREE Fraction

200 μL of serum was equilibrated at 37 °C for 30 min. The sample was transferred to an Microcon Ultracel PL-10, 10 kDa tubes (Millipore) and centrifuged at 12000xg and 37 °C for 30 min. 150 μL of the filtrate was then spiked with 20 μL of ISTD (^13^C_6_-T4, ^13^C_6_-T3, ^13^C_6_-rT3, ^13^C_6_-3,3′-T2, and ^13^C_6_-T1Am at 1 ng/mL in water) and vortexed. Next, 300 μL of 0.1% formic acid in acetonitrile was added. Subsequently, 150 μL of 35% NaCl was added, and the mixture was vigorously vortexed (20s). The mixture was centrifuged at 1900*g* and 4 °C for 10 min to achieve phase separation. The resulting supernatant was collected in amber glass conical vials and evaporated at 50 °C using a slow flow of nitrogen (30 psi, approximately 15 min).

### Derivatization

The yield of the derivatization reaction was assessed under various pH conditions (9, 10, 11), temperatures (40, 50, 60 °C), and reaction times (10–60 min). The analytes were labeled with dansyl chloride (dnsCl) using a 1:1 mixture of sodium bicarbonate/carbonate buffer (100 mM) and dnsCl (3 mg/mL in acetone). 200/100 μL (Total/Free fraction) of the mixture was added to the dry sample and vortexed (4 × 5s). Then, while avoiding light, the samples were incubated at 50 °C for 30 min. After derivatization, the samples were vortexed, cooled to 4 °C, and analyzed by HPLC-MS/MS.

### HPLC-ESI-MS/MS

Both, chromatographic separation and mass spectrometric detection were optimized with regard to selectivity, specificity, and sensitivity. An Agilent 1290 Infinity II (Agilent Technologies GmbH, Waldbronn, Germany) UPLC system including a binary solvent pump, a cooled autosampler (kept at 10 °C), and a column oven was used. Reversed phase (RP) separation was performed using a 2.1 mm × 100 mm, 1.7 μm particle, and pore size 130 Å, ACQUITY UPLC BEH C18 Column (Waters, Milford, MA, USA). The oven temperature was maintained at 30 °C. Solvents A and B were water (with 0.1% formic acid) and acetonitrile (with 0.1% formic acid), respectively. Ten microliters of each individual sample were injected for analysis. The following gradient elution program was utilized for chromatographic separation at a flow rate of 0.3 mL/min: 0–1 min 50–80% B; 1–4.5 min 80% B; 4.5–4.51 min 80–100% B; 4.51–6.50 min 100% B; and finally, 3 min of equilibration at 50% B.

An Agilent 6495 triple quadrupole mass spectrometer equipped with an electrospray ionization (ESI) source was operated in the positive ion mode using multiple reaction monitoring (MRM). The parameters of electrospray in the positive ion mode were capillary voltage, 3 kV; nozzle voltage, 2000 V; gas flow, 20 L/min; nebulizer pressure, 20 psi; sheath gas flow, 12 L/min; source temperature, 120 °C; and sheath gas temperature, 200 °C. The cycle time was set to 1000 ms, the fragmentor to 380 V, and the cell accelerator to 4 V. SRM transitions and collision energies were optimized for each analyte (Table S3).

### Quality Assurance

The method was validated for selectivity, recovery, matrix effect, linearity, trueness, and precision according to the guidelines of the Commission Decision 2002/657/EC.^28^ Selectivity was evaluated by analyzing six different lots of samples (blanks) and fortified samples to ensure the absence of other substances that might interfere with the targeted analytes and the ISTD in the samples. In all calculations, endogenous peak areas (nonspiked QC samples) corresponding to the selected compounds were subtracted. Extraction recovery was assessed by comparing the relative response (area of analyte/area of ISTD) obtained from extracted QC samples with matrix spiked post extraction, at three concentration levels. Precision was calculated as the coefficient of variation (CV) of the measurements. Both trueness and precision were investigated on three levels: high, medium, and low (blank matrix without spiking). The measurements were performed in 1 day (intraday, 5 replicates) and over 5 consecutive days (interday). The acceptable trueness values should be ≤ ± 20% and the CV characterizing the precision should not be higher than 15% (20% for the low-level).

The impact of the matrix on the signals of analytes was assessed by spiking native standards into extracts of the human serum. The peak areas of THs and THMs in the spiked samples were compared with those of their standards in water at equivalent concentrations. As endogenous analytes were naturally present in the serum sample, the peak areas found in the nonspiked sample were subtracted from those in the spiked samples enabling the calculation of actual matrix effects on the signals of THs. The experiment was conducted in quadruplicate. Matrix effects were expressed as the percentage ratios of peak areas of the serum extract to those of the water solution. To check the performance of the validated method, the matrix-matched reference material was prepared using a separate set of CRM standards (certified under ISO 17034, ISO/IEC 17025, and ISO 9001) and T3/T4 depleted the human serum. Details on reference material preparation are provided in the Supporting Information and Table S2.

### Subjects

In the current study, serum samples from 120 pregnant women enrolled in the CELSPAC:TNG cohort study were analyzed. The cohort represents the pregnant female population (third trimester). Cohort details are provided in the Supporting Information section.

## Results and Discussion

### Optimization of Derivatization Conditions

**Figure 1:**
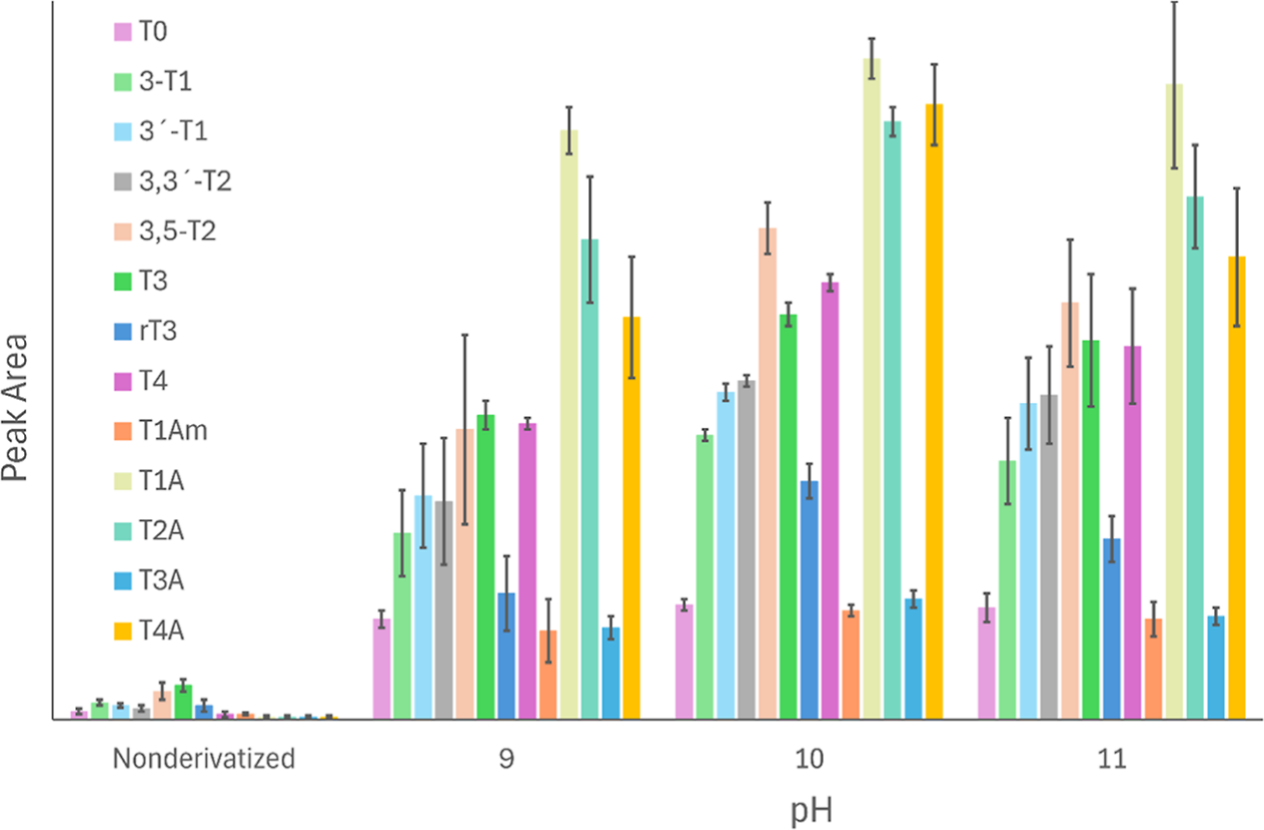
The comparison of signal intensities (given as peak areas) of non-derivatised analytes and analytes derivatised with dansyl chloride at different pH conditions. The experiments testing various pH levels showed significant differences in the dansylation yield for all analytes except T1A, T0 and T1Am (Abbreviations explained in Table 1). The highest yields were achieved, and the variability of the results was lowest at pH 10.

The effect of derivatization was assessed through a direct comparison of peak areas of neat and dansylated standards at 100 pg/mL. The tests with various temperatures (40–60 °C), pH values (9–11), and reaction times (10–60 min) to determine the optimal conditions for the dansylation^29−31^ showed the optimal yield for all THs and THMs at 50 °C, pH 10, with a reaction time of 30 min. Our results document that the dansylation enhanced sensitivity by more than 10-fold compared with the underivatized analytes (Figure 1). The increase in sensitivity was most prominent in the case of TAs. Figure S1 documents that nondansylated TAs at 500 pg/mL are close to the background, while dansylated analytes were clearly visible at 5 pg/mL. The degree of derivatization of respective TH and THM varies. While TAs are singly dansylated, the rest of the analytes containing both phenolic and amino groups are doubly labeled. This leads to different ion intensities in mass spectra and, thus, influences the detection limits.

### LC-MS/MS Method Selectivity, Sensitivity and Linearity

The dansylated analytes were separated using reversed phase HPLC and detected in the ESI positive mode-MS/MS (Figure S1B). The fragmentation of each analyte was examined to achieve the optimal sensitivity and selectivity. For all analytes, the dansyl fragments (*m*/*z* 171 and 156) were the most abundant product ions (Figure S2). Chromatographic separation of derivatized analytes was achieved in 8.0 min (Figure S1B) and enabled the baseline separation of the isobaric compounds (3,3′,5-T3/3,3′,5′-T3; 3,5-T2/3,3′-T2; and 3-T1/3′-T1). Retention time, two MRM transitions, and the relative response of the qualification/quantification transitions were employed for the definitive identification of the analytes.^32^Table S3 summarizes the MS/MS parameters and multiple reaction monitoring transitions.

Analyte responses were linear in a broad concentration range, as confirmed with a coefficient of determination (*r*^2^) value above 0.99 for all THM. Sensitivity was characterized by the methodological limit of quantification (MQL). For this, the depleted T3/T4 serum matrix extract was spiked with low concentrations (2–20 pg/mL, T4 250 pg/mL) of the target analytes, dansylated and analyzed as detailed above. Variability expressed as the standard deviation (*n* = 6) was used for MQL calculations (Table 1). The MQLs were determined as the concentration corresponding to a minimum signal-to-noise ratio of 10:1 observed in the sample matrix.^10,33^

### Sample Preparation

#### Total Fraction

**Figure 2:**
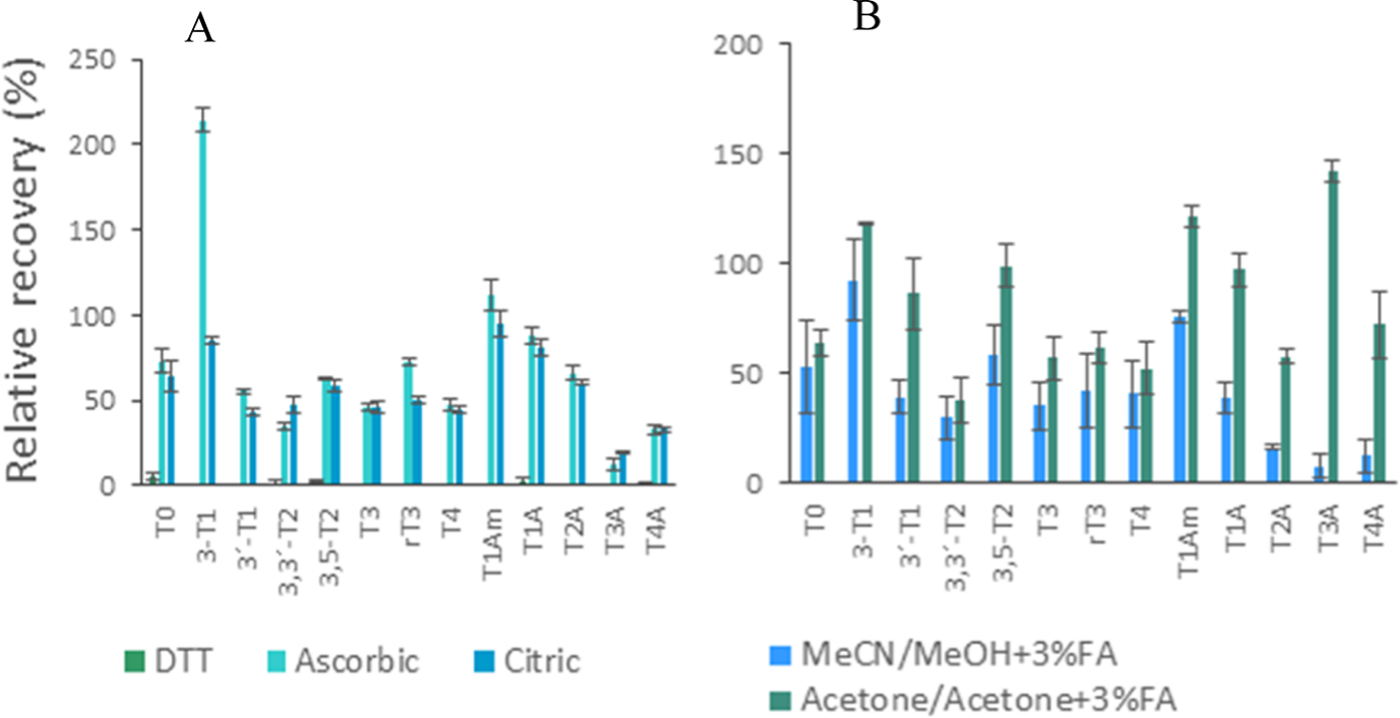
(A) The influence of different antioxidants (1,4-dithiothreitol, ascorbic, and citric acids) on relative recovery of THs and THMs. DTT decreased relative recovery of all analytes below 5%. Ascorbic acid increased concentrations of T1Am and 3-T1 (relative recovery > 200%) most probably due to the chemical reduction of abundant THs (T3, T4). (B) The influence of solvents used for protein precipitation and SPE elution step on relative recovery of THs and THMs. Acetone provides better recovery and lower variability due to higher volatility and better derivatization compatibility.

The general strategy of the total fraction preparation procedure was based on the method published by Jongejan et al.^17^ However, we identified a few problematic points that can strongly compromise the results. The proposed use of the antioxidant mixture (dithiothreitol, l-ascorbic, and citric acid, 1.75 mg/sample each) resulted in unpredictable effects on the recovery. We assessed the influence of its single constituents and found out that dithiothreitol (DTT) decreased the relative recovery of all analytes below 5%. On the other hand, l-ascorbic acid increased amounts of T1Am and 3-T1 (relative recovery >200%) most probably due to chemical reduction of abundant T3 and T4. With the addition of citric acid, the relative recoveries of all TH/THM were the closest to 100% with the lowest SDs (Figure 2A).

Different SPE sorbents that were previously employed for TH/THM were tested. Namely the mixed-polymeric OASIS HLB,^7,24^ the OASIS MCX cation-exchanger^25^ and OASIS MAX anion-exchanger. Jongejan et al.^17^ obtained good results with OASIS MAX when analyzing T3A and T4A. Thus, we decided to continue our tests following their approach. Unfortunately, namely, the results for T1A and T2A were not satisfactory with this strategy. To improve recoveries, we have employed ACE for protein precipitation, included a washing step with 5% NH_4_OH, and changed formic acid content in MeOH (to 3% FA) for elution. We noticed good recoveries but a higher variability of the results. The evaporation of the solvent before the derivatization step is crucial. The formic acid needs to be evaporated completely as the dansylation is pH dependent. After the evaporation step, when using formic acid in methanol, some of the dried samples had a very low pH and the traces of methanol severely affected dansylation. We switched the elution solvent to 3% formic acid in ACE, and the repeatability has improved dramatically (Figure 2B). Because the MAX cartridges work with the anionic exchange mode, the difference in polarity between MeOH and ACE did not negatively affect the recoveries.

Table 2 shows the recoveries assessed at two different spike levels (medium, high) before extraction by comparing the relative responses (area/area of ISTD) with those obtained through postextraction spiking. As we used a matrix blank (T3/T4 depleted human serum) that contains endogenous levels of the hormones, we included this level as the lowest, calculating trueness and precision based on it. We achieved very good recoveries for all analytes in the two spiked concentration ranges (84–119%). Slightly lower recovery (77%) was observed on a “High” spiking level for 3,3′-T2. However, this value is still satisfactory and can be corrected using the internal standard ^13^C_6_-3,3′-T2.

**Table 2:** Recoveries, Precision, and Matrix Effect of the TOTAL Fraction Method

parameter		T0	3-T1	3′-T1	3,3′-T2	3,5-T2	T3	rT3	T4	T1Am	T1A	T2A	T3A	T4A
low level	(pg/mL)	3.40	41.0	1.54	0.75	3.42	0.78	2.22	259	32.7	1.9	1.99	19.8	0.89
	intraday (CV, %)	2.55	3.99	2.29	8.74	2.45	0.93	1.90	1.66	4.21	4.92	13.2	9.21	3.30
	interday (CV, %)	10.0	7.05	6.26	45.0	4.65	1.69	2.68	2.62	10.7	27.7	35.4	35.6	33.6
medium	(pg/mL)	16.0	16.0	16.0	16.0	16.0	320	160	12,800	16.0	16.0	16.0	16.0	16.0
	recovery (%)	95.9	113	91.6	107	110	90.0	94.2	96.8	95.0	87.9	97.5	116	110
	intraday (CV, %)	2.09	3.07	5.34	3.87	2.50	4.01	1.85	1.73	3.86	1.66	6.31	5.19	1.30
	interday (CV, %)	6.68	5.50	3.98	12.1	2.70	3.26	1.78	1.29	7.31	3.61	10.5	21	13.9
high	(pg/mL)	40.0	40.0	40.0	40.0	40.0	800	400	32,000	40.0	40.0	40.0	40.0	40.0
	recovery (%)	91.7	119	91.5	77	109	111	111	109	111	94.1	84.2	104	118
	intraday (CV, %)	1.76	2.70	2.05	6.54	2.08	1.70	1.72	1.63	3.06	3.15	3.96	3.75	2.45
	interday (CV, %)	10.7	6.89	3.39	17.6	4.40	2.31	2.06	1.50	4.90	2.86	3.40	11.7	7.86
matrix effect	mean, %	–15.5	–15.3	–7.8	–15.9	–11.7	–0.7	9.9	6.3	15.5	–27.5	–41.0	–13.4	–6.9
	CV, %	4.1	1.7	5.9	3.1	4.2	3.4	4.1	5.1	6.7	4.7	8.0	3.6	2.2

Matrix effects were evaluated by comparing signal intensity in the matrix spiked post extraction and standard solution in water. Positive and negative values represent ion enhancement and ion suppression, respectively. Matrix effects were below ±16%, except for T1A and T2A, and with CV < 8% (Table 2). Ion suppression was observed in the case of 3′-T1, 3,5-T2, T0, 3-T1, 3,3′-T2, T3, and TAs, while the rest of the analytes showed ion enhancement. For the internal standards the matrix effects were also evaluated (data not shown), obtaining results ±15% with CV > 7%. The matrix effects in the case of T1A and T2A are slightly higher (−27.5 and −41%). We tested different approaches, including LLE, salt-out partitioning, and SPE, on the reverse phase as well as ionic and mixed-mode sorbents. However, the methods presented in the manuscript provided the best overall results.

We used intra- and interday variations to evaluate the precision of the method. The intraday CVs ranged 0.93–13.2%. Interday CVs for almost all target analytes ranged from 1.29 to 13.8%. Some analytes in the low-level (3,3′-T2, T1A, T2A, T3A, T4A) showed higher interday variability (27.7–45%), but this is mainly due to the low concentrations close to/on the level of MQL and thus higher measurement uncertainty (Table 2). The data were measured with no special care or cleaning done on the instrument and thus reflect the standard rather than the ideal performance of the method and system, which are prone to instrumental drift and fluctuations.

#### Free Fraction

**Figure 3:**
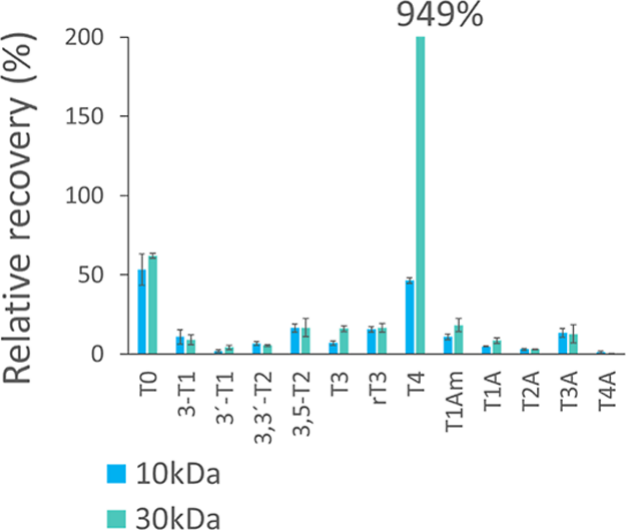
Comparison of Microcon Ultracel PL-10 (10 kDa) and Amicon Ultra-0.5 (30 kDa) ultrafiltration devices—permeability of the UF membrane for THs and THMs in the spiked human serum. Relative recoveries of THs and THMs are generally lower than the spiked amount (100%) due to the equilibria shift in the serum. Relative recovery of T4 was 1 order of magnitude higher (+949%) than expected when 30 kDa unit was used.

**Figure 4:**
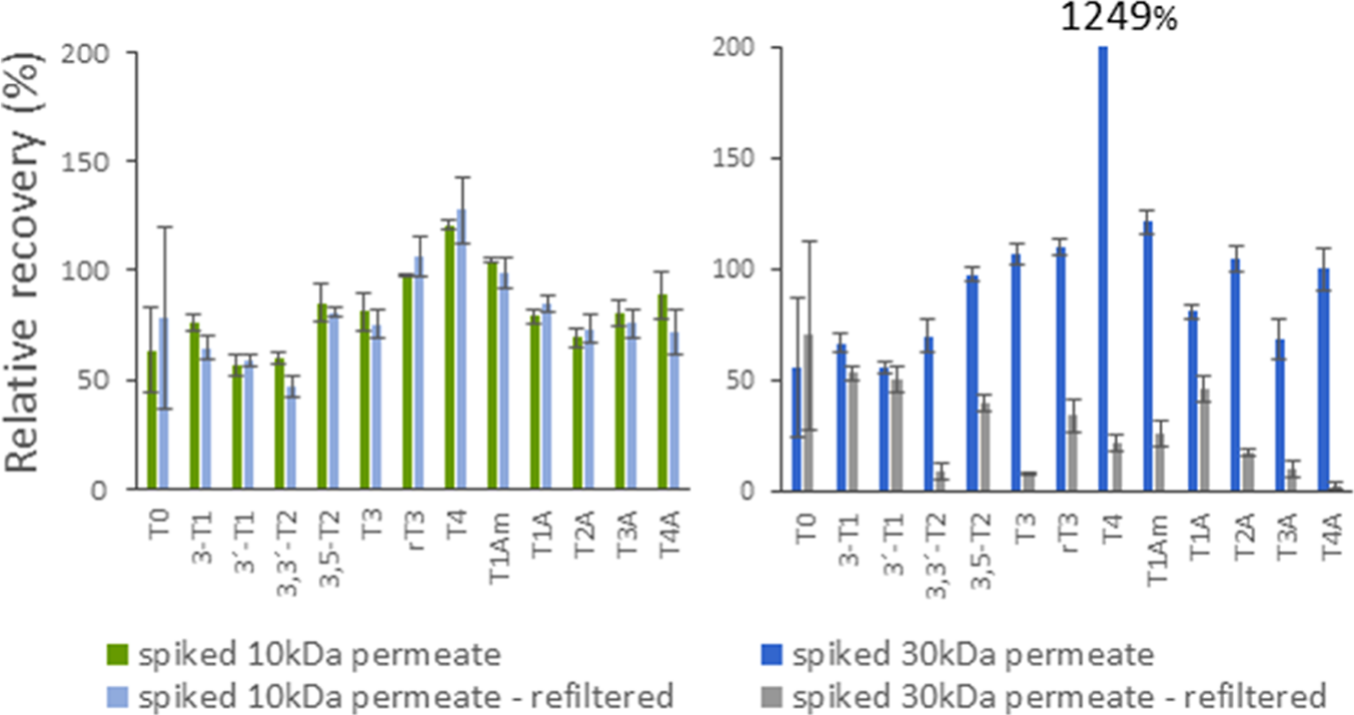
Effect of repeated ultrafiltration step on the levels of THs and THMs in the spiked human serum. 30 kDa filtration device (Amicon Ultra-0.5, 30 kDa) exhibits significant decrease (5–95%) in most target analyte concentrations, whereas the difference for 10 kDa unit (Microcon Ultracel PL-10) is negligible.

The general strategy of the FREE fraction sample preparation was based on ultrafiltration methods. Based on previous studies,^24^ we have compared two commonly used ultrafiltration membranes (Amicon Ultra-0.5, 30 kDa; Microcon Ultracel PL-10, 10 kDa). When using ultrafiltration through 30 kDa membranes (Amicon Ultra-0.5), we observed unusually high levels of T4 (up to 1 order of magnitude higher than expected, Figure 3). This corresponds to the leakage of a fraction of protein-bound T4 through certain ultrafiltration devices observed in some previous studies^24^ and discussed in a recent mini-review on free TH analyses.^26^ On the other hand, when the ultrafiltration step was repeated with spiked permeate from ultrafiltration, the relative recoveries of the rest of TH and THM with Amicon 30 kDa membranes were decreasing (Figure 4). This finding is consistent with Fritz et al.,^27^ who observed T3 and T4 losses with Amicon Ultra-4 (10 kDa). Moreover, these effects cannot be taken into account/quantified by means of the isotope dilution method. Thus, to minimize any biases and ensure optimal separation of free hormones from those bound to plasma proteins, we further employed 10 kDa cellulose membrane filters Microcon Ultracel PL-10 that showed reproducible recoveries of analytes and no excessive migration of T4 through the device.

Due to the limited amount of the sample and very low concentrations (sub ppt) of target analytes in the free fraction, we decided to minimize sample preparation steps and thus avoid a SPE cleanup. Salt-out liquid partitioning was finally chosen as the most appropriate method that allowed minimal handling and offered dansyl derivatization compatibility, excellent recoveries, minimal interferences and, thus, a low chromatogram background, and reproducible quantification of very low-abundance THM like T2A, T3A, and T1Am, analytes typically reported as nondetected in other studies (Table 4).

Table 3 shows the recoveries evaluated at two different spike levels before extraction, comparing the relative responses (area/area of ISTD) with spiking postextraction. As in the TOTAL fraction, we used a matrix blank (T3/T4 depleted human serum). However, the concentrations for most of the analytes in its FREE fraction were below the LOD/LOQ and the calculation of the trueness and precision was not possible. That is why the data are available for LOW and HIGH spiked levels only. We obtained very good recoveries (73–115%) for all of the analytes in two physiologically relevant concentration ranges in pg/mL levels.

**Table 3:** Recoveries, Precision, and Matrix Effect of the FREE Fraction Method

parameter		T0	3-T1	3′-T1	3,3′-T2	3,5-T2	T3	rT3	T4	T1Am	T1A	T2A	T3A	T4A
low level	(pg/mL)	3.0	3.0	3.0	3.0	3.0	3.0	3.0	3.0	3.0	3.0	3.0	3.0	3.0
	recovery (%)	77.4	92.4	90.7	111.5	99.7	95.0	116	99	100.5	99.7	114.1	72.7	102.1
	intraday (CV, %)	8.8	5.9	16.4	9.6	7.1	4.9	6.9	1.5	8.5	7.8	8.7	7.6	4.1
	interday (CV, %)	9.8	6.2	13.6	14.9	9.4	5.4	22.6	5.0	10.0	8.2	9.1	22.9	14.7
high level	(pg/mL)	9.0	9.0	9.0	9.0	9.0	9.0	9.0	9.0	9.0	9.0	9.0	9.0	9.0
	recovery (%)	95.9	96.6	93.2	104.8	101.8	104	108	101	114.8	95.1	102.4	94.1	112.7
	intraday (CV, %)	2.8	7.5	2.9	1.5	4.1	1.8	2.6	1.2	1.5	6.8	3.5	3.7	3.2
	interday (CV, %)	15.1	7.4	12.5	9.4	4.8	2.8	12.2	4.6	10.4	6.2	10.0	5.6	4.0
matrix effect	mean, %	–11.7	–11.4	–16.5	–9.9	–13.1	–24	–46.1	13	–5.6	–23	–26.5	–6.5	–23.7
	CV, %	3.1	1.8	6.1	3.3	6.3	6.9	9.0	15	8.4	6.7	3.1	6.6	6.3

The method to evaluate the matrix effects was analogous to the TOTAL fraction. Matrix effects were below ±25%, except for rT3 and T2A, and with CV ≤ 15% (Table 3). Ion suppression was observed for all analytes, except for T4, which showed slight ion enhancement. For the internal standards, the matrix effects (data not shown) ranged up to ±19%, with CV < 9%. Compared to the TOTAL fraction, the suppression of signal of rT3 is more pronounced. The FREE fraction samples contain more interferences due to the less elaborate cleanup procedure (compared to TOTAL fraction samples undergoing a more complex SPE cleanup). A similar pattern can be observed also for other TAs. Nevertheless, from a practical perspective, matrix effects are estimated values that exhibit some variability among real samples due to the unique interference composition of individual specimens. Moreover, the values provided in our manuscript represent rather a “worst-case scenario”, based on net IS nonadjusted peak areas at the “Low” spiking level.

Intra- and interday variations were used to evaluate the precision of the method. Intraday CVs ranged from 1.2 to 16.4%. Interday CVs for almost all target analytes ranged from 2.8 to 14.9% except for some analytes in the low-level (rT3, T3A).

### Application of Validated Methods

Normal thyroid hormone reference intervals in the human serum have a large degree of variability based on several factors including age, sex, etc. The samples in our study represent a pregnant female population (3rd trimester). The range is calculated based on the 2.fifth to 97.fifth percentile. We report herein our reference ranges and compare them with the latest studies found. It is important to note that this is the first time that 11 THM are reported along with T3 and T4 (Table 4) in the human serum. Moreover, to the best of our knowledge, our study provides the first information on the detected T1A and T2A levels in the human serum.

**Table 4:** Nonparametric Reference Concentration Intervals of the Total Fraction of Two Thyroid Hormones and 11 Metabolites in the Human Serum (*n* = 120 Pregnant Women, 3rd Trimester)

		mean	SD	2.5th percentile (90% CI)	25th percentile (90% CI)	median	75th percentile (90% CI)	97.5th percentile (90% CI)
T0	total (pg/mL)	43.9	13.9	23.2	36.15	42	48.4	81
	free (pg/mL)	8.71	3.13	4.53	6.49	8.05	9.99	17.60
3-T1	total (pg/mL)	14.5	7.70	<6.5	9.43	13.8	18.96	30.8
	free (pg/mL)	0.88	3.19	<0.3	<0.3	0.38	0.99	2.33
3′-T1	total (pg/mL)	2.24	1.60	0.47	1.23	1.94	2.88	6.11
	free (pg/mL)	<0.2	<0.2	<0.2	<0.2	<0.2	<0.2	0.73
3,3′-T2	total (pg/mL)	2.27	4.20	<0.4	<0.4	0.49	1.22	15.5
	free (pg/mL)	<0.2	0.24	<0.2	<0.2	<0.2	0.22	0.82
3,5-T2	total (pg/mL)	13.24	10.12	1.26	6.51	10.4	16.4	42.6
	free (pg/mL)	0.22	0.29	<0.1	<0.1	<0.1	0.40	0.94
T3	total (ng/mL)	1.02	0.18	0.71	0.88	1.01	1.15	1.36
	free (pg/mL)	1.63	0.86	0.70	1.07	1.48	2.00	4.18
rT3	total (pg/mL)	153.8	34.8	101.1	129.3	148.3	172.5	220.5
	free (pg/mL)	0.92	0.77	<0.4	<0.4	0.73	1.12	2.82
T4	total (ng/mL)	101.4	20.5	63.9	89.4	100.4	111.5	142.2
	free (pg/mL)	10.26	5.38	4.78	7.55	9.49	11.42	19.25
T1Am	total (pg/mL)	7.53	7.73	<4.6	<4.6	4.63	9.44	32.5
	free (pg/mL)	<0.5	<0.5	<0.5	<0.5	<0.5	<0.5	0.85
T1A	total (pg/mL)	3.25	3.21	0.22	1.57	2.06	4.64	11.2
	free (pg/mL)	<0.3	<0.3	<0.3	<0.3	<0.3	<0.3	<0.3
T2A	total (pg/mL)	1.58	2.13	<0.6	<0.6	0.67	1.92	8.86
	free (pg/mL)	0.30	0.29	<0.1	0.12	0.12	0.43	1.10
T3A	total (pg/mL)	10.7	10.9	<3.3	<3.3	7.63	16.8	34
	free (pg/mL)	<0.3	<0.3	<0.3	<0.3	<0.3	<0.3	1.21
T4A	total (pg/mL)	8.65	6.22	2.24	4.91	7.11	9.55	26.2
	free (pg/mL)	<0.2	<0.2	<0.2	<0.2	<0.2	<0.2	<0.2

Our reference ranges for the T4, T3, and rT3 are similar to those reported by recent studies.^16,17,25^ Regarding 3,5-T2 only the method by Lorenzini et al.^13^ had enough sensitivity to detect its concentrations in the human serum and our results are in the same magnitude range. In the case of 3,3′-T2, four studies reported serum concentrations for humans of various health status (none of them being pregnant women). Our results are in concordance with two of them,^16,17^ while two studies focused predominantly on thyroid patients^25,34^ presented higher values (27–400 pg/mL). 3-T1 levels in pregnant women varied from below MQL (6.5 pg/mL) to 30.8 pg/mL in our study. They are about 1 order of magnitude higher compared to the only other available study in adults (19–71y),^17^ where, however, all reported 3-T1 concentrations were below their listed limits of quantification of 8 pg/mL. Next, T0 interval ranging from 23.2 to 81 pg/mL was similar to the values reported by Jongejan et al.^17^ About 2–3 fold greater T0 levels were reported in 21 nonathletes in a recent study by Martínez Brito et al.,^35^ which, however, emphasized that their values were close to the LLOD or LOQ and should be considered estimated values. For T1Am, we found only one study^36^ that reports concentrations detected by LC–MS/MS using 1 mL of serum from 41 hospital patients and one healthy volunteer (mean age 58 yrs). The mean levels detected in pregnant women from our cohort (3rd trimester) are about an order of magnitude lower, while the greatest detected levels are close to that of a previous study. In the case of T3A, we did not find any other study with human serum levels determined by LC–MS/MS. Our detected levels for T3A are consistent with Menegay et al.,^37^ which reported T3A levels below their detection limit of 34 pg/mL across euthyroid, hypothyroid, and hyperthyroid serum samples using RIA and slightly lower than those summarized in a review^38^ of older literature (1955–1992), which often included patients with some thyroid disorders. Finally, the range of T4A levels detected in the pregnant women in the third trimester in our study reaches up to the lower levels reported in previous studies for other population groups.^17,35,39^

The literature data on the free fraction of thyroid hormones and namely their metabolites in the human serum determined by LC–MS/MS are rather limited. Table 4 presents reference ranges for the free fraction levels of thyroid hormone metabolites, along with fT3 and fT4, obtained using our newly developed method. As far as we know, only T3 and T4 free fraction levels from the LC–MS/MS methods have been published, and our data are in good agreement with the reported results.^11,40−42^ Our detected fT3 levels are within the range (0.3–6.5 pg/mL) published in the literature. Also the fT4 concentrations detected in our study correspond well to the reported range (7.8–31.1 pg/mL) determined by LC–MS/MS in the human serum.^11,40−42^ To the best of our knowledge, all other detected free THM levels in the human serum are reported for the first time in our study (Table 4).

In summary, the novel optimized methodology employing derivatization of TH and THM in the human serum has led to more than an order of magnitude increase in sensitivity, as well as enhanced selectivity. This is confirmed also by comparison with the comprehensive summary of the parameters of LC–MS/MS methods currently used for the determination of thyroid hormones and their metabolites compiled in the recent review of Jin et al.^43^ The increase in sensitivity was most prominent in the case of TAs. Our methods (Table 1) are more sensitive than, e.g., those reported by Hansen et al.^10^ for T4 (233 vs 70.6 pg/mL), T3 (96.7 vs 1.1 pg/mL), and T1Am (110 vs 4.61 pg/mL), respectively. Kiebooms et al.^7^ obtained a similar MQL for T4 (40 pg/mL), but higher MQLs for T3 (30 pg/mL) and rT3 (50 pg/mL) than our method, even when they used 1000 mL of the serum, which in some cohorts like newborns is a prohibitive amount.

## Conclusions

This study established sensitive and robust high throughput LC–MS/MS methods for the analysis of a broad range of thyroid hormones and their metabolites in the human serum. For the first time, the derivatization of a broad spectrum of TH and THM in the human serum is described, leading to enhanced sensitivity and selectivity. Our methods provide excellent sensitivity, reliability, and robustness, confirming the suitability of the optimized sample preparation procedure for TH/THM analysis in the human serum. The applicability of the method was evaluated on serum samples from the CELSPAC:TNG cohort to validate the suitability of our method for the routine high throughput analysis of TH and metabolites. Thus, our results also bring the first information on most THM in pregnant women. The obtained results are encouraging, as the detected concentration levels of TH and metabolites in the serum are in ranges comparable to those reported in the literature. This method could have a significant impact in the field of hormone analysis because it enables the detection of a wide spectrum of TH metabolites, including several that were undetectable by previous methods. To the best of our knowledge, it is for the first time that the data for free THM levels in the human serum are reported in the literature. We expect that our method could contribute to reveal biological functions of analytes (e.g., T0, T2A, 3-T1), where the knowledge is severely limited.

## Supporting Information Available

Molecular structures of analyzed thyroid hormones and metabolites; description of study subjects; TH and THM chromatographic separation and fragmentation patterns; detailed MS/MS conditions; and supporting references (PDF)

## Notes

Compliance with ethical standards Ethical approval: study name—Central European Longitudinal Study of Parents and Children: The Next Generation (acronym: CELSPAC: TNG, ethics committee: Multicenter and Local Ethical Committee of University Hospital Brno, ref no. 20140409-01, date: 09/04/2014).

## Notes

Informed consent: The Multicenter and Local Ethical Committee of University Hospital Brno, the Czech Republic, approved the CELSPAC: TNG study (ref. no. 20140409-01, date 09/04/2014). All pregnant women gave their written informed consent.
