# Supplementary material for: Determination of Thyroid Hormones and 11 Metabolites in the Human Serum Using a Simple Derivatization Strategy and Analysis by Isotope-Dilution Liquid Chromatography Tandem Mass Spectrometry
Source: Anal Chem. 2025 Apr 23;97(17):9438–46. doi: 10.1021/acs.analchem.5c00714 (PMC12060091; doi:10.1021/acs.analchem.5c00714)
Supplement: Supplementary file 1 — ac5c00714_si_001.pdf [file ac5c00714_si_001.pdf]

Supporting Information

Determination of thyroid hormones and 11 metabolites in human serum using a simple derivatization strategy and analysis by isotope-dilution liquid chromatography tandem mass spectrometry

Jiří Kohoutek [a], Juan I. Sánchez-Avila [a,b], Marie Smutná [a], Petr Janků [c,d], Jana Klánová [a] and Klára Hilscherová[a]*

[a] RECETOX, Faculty of Science, Masaryk University, Kotlarska 2, 602 00 Brno, Czech Republic

[b] CeMM Research Center for Molecular Medicine of the Austrian Academy of Sciences, Lazarettgasse 14, AKH BT 25.3, 1090 Vienna, Austria

[c] Clinic of Gynecology and Obstetrics, University Hospital Brno, Jihlavska 20, 625 00 Brno, Czech Republic

[d] Department of Health Sciences, Faculty of Medicine, Masaryk University, Kamenice 126/3, 625 00 Brno, Czech Republic

Table of Content

Table S1. Thyroid hormones and metabolites included in this study, their molecular structure and reported effects or biological activity.	S2
Subjects	S5
Reference Material Preparation	S5
Table S2. TH and THM concentrations in standard mixture used for reference material preparation	S5
Figure S1. TIC chromatogram of the thyroid hormones (THs) and 11 thyroid hormone metabolites (THMs)	S6
Figure S2. Mass spectrum of the dansylated T1A and the predicted fragmentation pattern for dansylated T1A and T3	S7
Table S3. Optimized MRM conditions for the identification (*Q*) and quantification (*q*) of the dansylated (dns-) THs and 11 THMs	S8
Supporting References	S9

Table S1. Thyroid hormones and metabolites included in this study, their molecular structure and reported effects or biological activity.

Analyte (abbreviation)	Molecular structure	Reported effects or biological activity
Thyronine<br>(T0)	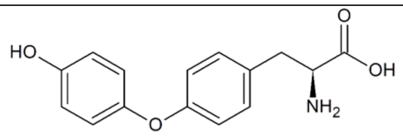	Biological activity is unknown.<br>It has been detected as deiodinated product of 3-T1 in cell lysates after incubation [1]
3-iodothyronine<br>(3-T1)	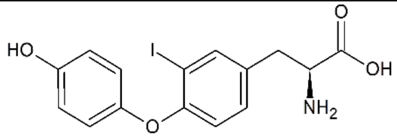	− Biological activity is unknown
3’-iodothyronine<br>(3′-T1)	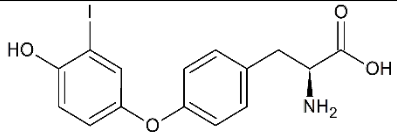	− Biological activity is unknown.
3,3’-diiodothyronine<br>(3,3'-T2)	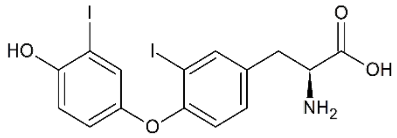	Reported relation between higher 3,3′-T2 concentrations in athyreotic outpatients receiving thyroid stimulation hormone suppression therapy [2].<br>− Diiodothyronines in the range of plasma physiological concentrations reduced hepatic lipid accumulation. They act as regulators of hepatic metabolic homeostasis [3].
3,5-diiodothyronine<br>(3,5-T2)	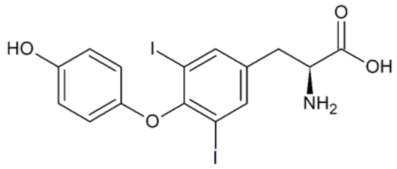	Increases metabolic rate, reduces body weight gain, cholesterol and triglycerides levels, hepatic steatosis, insulin resistance and blood glucose[4–6].<br>Rapid stimulatory metabolic effects on mitochondrial respiration and oxygen consumption have been demonstrated in mostly hypothyroid rodent models [7].
3,3’,5-triiodothyronine<br>(T3)	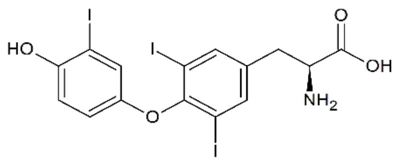	− Two to four times more active than T4 [8]<br>− Influence in:<br>● Growth and development [9,10]<br>● Carbohydrate and lipid metabolism [9,10]<br>● Oxygen consumption [9,10]<br>● Protein synthesis [9,10]<br>− Fetal neurodevelopment [9,10]
3,3’,5’-triiodothyronine<br>(rT3)	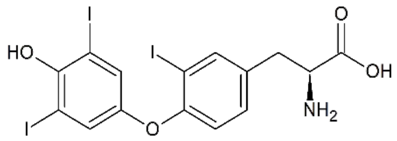	Inhibits thyroid production of T3 through feedback mechanisms [9,11].<br>Non-genomic effects on for example actin remodelling and brain development [11]

		Probable host factor supporting cancer growth [12].<br>− rT3/T3 concentration ratio may also be a useful diagnostic biomarker for evaluating the function of deiodinase enzymes [13].
3,3’,5,5’-tetraiodo-L-thyronine or<br>L-thyroxine<br>(T4)	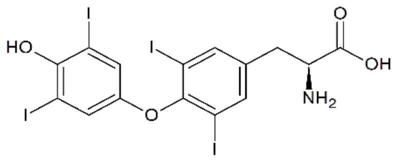	− Prohormone [9,10]<br>− Influence in:<br>● Growth and development [9,10]<br>● Carbohydrate and lipid metabolism [9,10]<br>● Oxygen consumption [9,10]<br>● Protein synthesis [9,10]<br>● Fetal neurodevelopment [9,10]<br>● Cell proliferation and angiogenesis [14]
3-iodothyronamine<br>(T1Am)	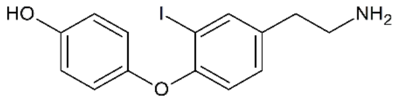	Metabolic, anapyrexic, cytoprotective, and brain effects; influences cardiac function and glucoregulatory processes, resulting in hypoinsulinemia, hyperglucagonemia and hyperglycemia. [15–17].<br>Administration to experimental animals induces a “torpor-like” state [5].<br>Improves anti-amnesic and learning capacity [12].<br>Lipolytic and thermoregulation effects [12].<br>Decreases growth and viability of cancer cells [12].
3-iodothyroacetic acid<br>(T1A)	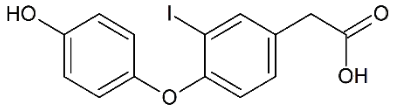	Studies in rats reported that this is a product of the T1AM [18,19]<br>Behavioural and metabolic effects; stimulation of memory, reduction of pain threshold and increase of plasma glycaemia in rats [19]<br>Works as a potential activator of waking and learning [20]
3,5-diiodothyroacetic acid<br>(T2A)	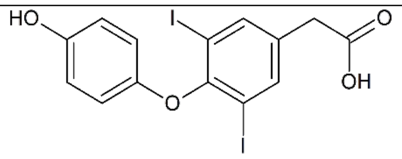	Biological activity is unknown, but evidence suggests that thyronamines are oxidatively deaminated to iodothyroacetic acids in vivo [18].
3,3’,5-triiodothyroacetic acid<br>(T3A; Triac)	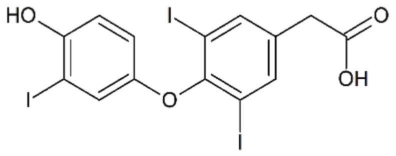	Thyromimetic - binds TH receptor with a similar/higher affinity than T3 [21]<br>Effects on brain development, TH signaling and brain network function. [12,22]<br>Reduces expression and secretion of leptin; exhibits anti-inflammatory properties [12].

3,3',5,5'-tetraiodothyroacetic acid<br><br>(T4A; Tetrac)	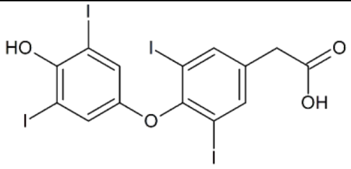	Anti-proliferative, pro-apoptotic and anti-angiogenic effect in cancer cells [4,12]<br><br>Increases brain development [4,12]

Experimental section

Subjects

In the current study, serum samples from 120 pregnant women enrolled in the CELSPAC: TNG cohort study were analysed. The cohort represents pregnant female population (3rd trimester). The CELSPAC: TNG study (Central European Longitudinal Studies of Parents and Children: The Next Generation) is designed as a prospective birth cohort, following pregnant mothers and their children from the prenatal period through to adolescence, with the aim of assessing exposome factors that may influence child health and development. The CELSPAC: TNG study builds on previous efforts of Masaryk University, in particular the Czech part of the WHO-initiated European Longitudinal Study on Pregnancy and Childhood (ELSPAC) [23]. The Multicentre and Local Ethical Committee of University Hospital Brno, the Czech Republic, approved this study (Ref. No. 20140409-01, date 09/04/2014). All pregnant women gave their written informed consent.

Reference Material Preparation

The matrix matched reference material was prepared using a separate set of CRM standards of T3, rT3, T4 (Cerilliant, Merck KGaA) and analytical standards of THM. 200 µL of standard mixture (Table S2) was added into 20 mL of T3/T4 depleted human serum (BBI Solutions). The enriched serum was homogenized and kept for 30 minutes at 37 °C to establish the total/free fraction equilibrium. Subsequently, the resulting material was aliquoted and frozen. The reference material was further stored at -80 °C.

Table S2. TH and THM concentrations in standard mixture used for reference material preparation

Analyte	T0	3-T1	3´-T1	3,3´-T2	3,5-T2	T3	rT3	T4	T1Am	T1A	T2A	T3A	T4A
ng/mL	1.6	1.6	1.6	1.6	1.6	32.0	16.0	1280.0	1.6	1.6	1.6	1.6	1.6

Results

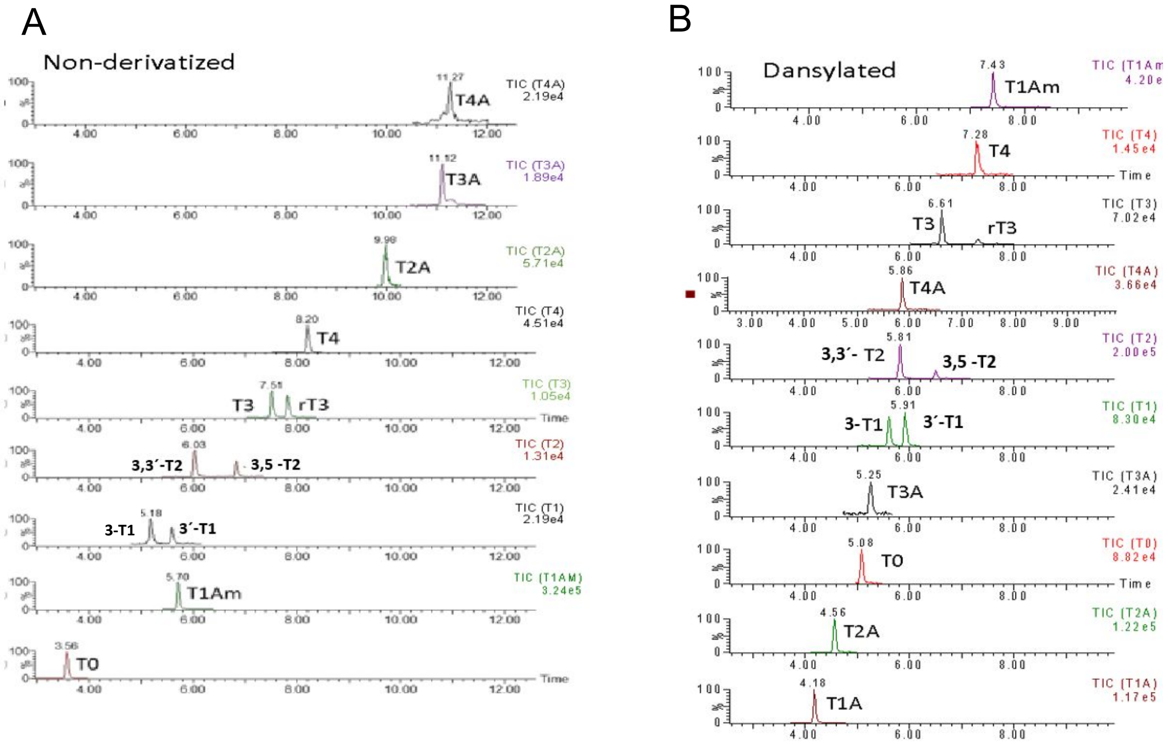

Fig.S1. TIC chromatogram of the thyroid hormones (THs) and 11 thyroid hormone metabolites (THMs). A) Non-derivatized analytes; the thyroacetic acids (in negative mode) are at 500 pg/mL and the rest (in positive mode) at 200 pg/mL; T1A was not detected. B) dansylated analytes, all analyzed in positive ESI mode at a concentration of 50 pg/mL.

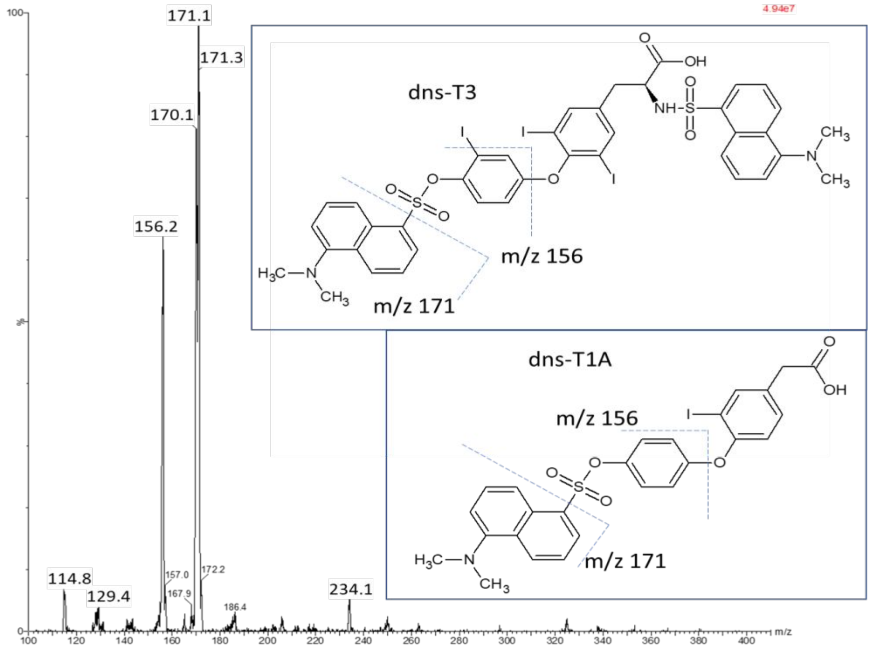

Fig.S2. Mass spectrum of the dansylated T1A and the predicted fragmentation pattern for dansylated T1A and T3

Table S3. Optimized MRM conditions for the identification (*Q*) and quantification (*q*) of the dansylated (dns-) THs and 11 THMs. The q/Q ratio was used for positive identification and the internal standards for quantification.

Compound Name	MRM transition of quantification (*Q*)	Collision Energy (V)	MRM transition of confirmation (*q*)	Collision Energy (V)	Rel. Response (q/Q, %)	Ret. Time (min)	Internal standard used
T0	370.6<171.0 (*Q*)	25	370.6<156.0	55	30.0	5.08	$^{13}C_6$-3,5-T2
3-T1	433.6<171.1 (*Q*)	21	433.6<156.0	45	36.9	5.72	$^{13}C_6$-3,5-T2
3´-T1	433.6<171.1 (*Q*)	21	433.6<156.0	45	40.5	5.91	$^{13}C_6$-3,5-T2
3,3´-T2	496.5<156.1 (*Q*)	41	496.5<171.1	25	338.5	5.81	$^{13}C_6$-3,5-T2
3,5-T2	496.5<171.1 (*Q*)	25	496.5<156.1	41	29.5	6.43	$^{13}C_6$-3,5-T2
T3	559.5<171.1 (*Q*)	25	559.5<156.0	52	24.2	6.61	$^{13}C_6$-T3
rT3	559.5<171.0 (*Q*)	25	559.5<155.8	57	23.2	7.16	$^{13}C_6$-rT3
T4	622.4<171.1 (*Q*)	25	622.4<155.8	60	21.1	7.28	$^{13}C_6$-T4
T1Am	411.6<171.1 (*Q*)	25	411.6<156.0	55	31.0	7.43	$^{13}C_6$-T1Am
T1A	604.0<171.1 (*Q*)	37	604.0<156.0	60	46.8	4.18	$^{13}C_6$-3,5-T2
T2A	729.9<171.0 (*Q*)	37	729.9<156.1	57	34.0	4.56	$^{13}C_6$-3,5-T2
T3A	855.8<171.1 (*Q*)	37	855.8<156.0	49	18.0	5.25	$^{13}C_6$-T3
T4A	981.7<170.9 (*Q*)	29	981.7<155.9	45	19.6	5.86	$^{13}C_6$-T4
$^{13}C_6$-3,5-T2	499.5<156.1 (*Q*)	41	499.5<171.1	25	30.2	6.42	---
$^{13}C_6$-T3	562.5<171.1 (*Q*)	21	562.5<156.1	53	25.2	6.60	---
$^{13}C_6$-rT3	562.5<171.1 (*Q*)	21	562.5<155.9	53	25.2	7.14	---
$^{13}C_6$-T4	625.4<171.1 (*Q*)	29	625.4<155.8	60	22.4	7.28	---
$^{13}C_6$-T1Am	414.6<171.1 (*Q*)	21	414.6<156.1	29	24.0	7.41	---

SUPPORTING REFERENCES

1. Richards KH, Schanze N, Monk R, Rijntjes E, Rathmann D, Köhrle J. A validated LC-MS/MS method for cellular thyroid hormone metabolism: Uptake and turnover of mono-iodinated thyroid hormone metabolites by PCCL3 thyrocytes. *PloS One*. 2017;12(8):e0183482. doi:10.1371/journal.pone.0183482

2. Jonklaas J, Sathasivam A, Wang H, et al. 3,3'-Diiodothyronine Concentrations in Hospitalized or Thyroidectomized Patients: Results from a Pilot Study. *Endocr Pract*. 2014;20(8):797-807. doi:10.4158/EP13453.OR

3. Gnocchi D, Ellis ECS, Johansson H, et al. Diiodothyronines regulate metabolic homeostasis in primary human hepatocytes by modulating mTORC1 and mTORC2 activity. *Mol Cell Endocrinol*. 2020;499:110604. doi:10.1016/j.mce.2019.110604

4. Hoefig CS, Zucchi R, Köhrle J. Thyronamines and Derivatives: Physiological Relevance, Pharmacological Actions, and Future Research Directions. *Thyroid*. 2016;26(12):1656-1673. doi:10.1089/thy.2016.0178

5. Glossmann HH, Lutz OMD. Torpor: The Rise and Fall of 3-Monoiodothyronamine from Brain to Gut—From Gut to Brain? *Front Endocrinol*. 2017;8:1-9. doi:10.3389/fendo.2017.00118

6. Lehmphul I, Hoefig CS, Köhrle J. 3-Iodothyronamine reduces insulin secretion in vitro via a mitochondrial mechanism. *Mol Cell Endocrinol*. 2018;460:219-228. doi:10.1016/j.mce.2017.07.026

7. Köhrle J. Thyroid Hormones and Derivatives: Endogenous Thyroid Hormones and Their Targets. In: Plateroti M, Samarut J, eds. *Thyroid Hormone Nuclear Receptor: Methods and Protocols*. Springer; 2018:85-104. doi:10.1007/978-1-4939-7902-8_9

8. Chopra IJ. Description, History, and Calorigenic Activity of Iodothyronines. In: *Triiodothyronines in Health and Disease. Monographs on Endocrinology*. SPRINGER; 1981.

9. Wang D, Stapleton HM. Analysis of thyroid hormones in serum by liquid chromatography-tandem mass spectrometry. *Anal Bioanal Chem*. 2010;397(5):1831-1839. doi:10.1007/s00216-010-3705-9

10. Bianco AC, Salvatore D, Gereben B, Berry MJ, Larsen PR. Biochemistry, cellular and molecular biology, and physiological roles of the iodothyronine selenodeiodinases. *Endocr Rev*. 2002;23(1):38-89. doi:10.1210/edrv.23.1.0455

11. van der Spek AH, Fliers E, Boelen A. The classic pathways of thyroid hormone metabolism. *Mol Cell Endocrinol*. 2017;458:29-38. doi:https://doi.org/10.1016/j.mce.2017.01.025

12. Senese R, Cioffi F, Petito G, Goglia F, Lanni A. Thyroid hormone metabolites and analogues. *Endocrine*. 2019;66(1):105-114. doi:10.1007/s12020-019-02025-5

13. Tanoue R, Kume I, Yamamoto Y, et al. Determination of free thyroid hormones in animal serum/plasma using ultrafiltration in combination with ultra-fast liquid chromatography-tandem mass spectrometry. *J Chromatogr A*. 2018;1539:30-40. doi:10.1016/j.chroma.2018.01.044

14. Mullur R, Liu Y yun, Brent GA. Thyroid hormone regulation of metabolism. *Physiol Rev*. 2014;94(27):355-382. doi:10.1152/physrev.00030.2013

15. Piehl S, Hoefig CS, Scanlan TS, Köhrle J. Thyronamines—Past, Present, and Future. *Endocr Rev*. 2011;32(1):64-80. doi:10.1210/er.2009-0040

16. Lehmphul I, Hoefig CS, Köhrle J. 3-Iodothyronamine reduces insulin secretion in vitro via a mitochondrial mechanism. *Mol Cell Endocrinol*. 2018;460:219-228. doi:10.1016/j.mce.2017.07.026

17. Köhrle J, Biebermann H. 3-Iodothyronamine—A Thyroid Hormone Metabolite With Distinct Target Profiles and Mode of Action. *Endocr Rev*. 2019;40(2):602-630. doi:10.1210/er.2018-00182

18. Wood WJLL, Geraci T, Nilsen A, DeBarber AE, Scanlan TS. Iodothyronamines are Oxidatively Deaminated to Iodothyroacetic Acids in vivo. *ChemBioChem*. 2009;10(2):361-365. doi:10.1002/cbic.200800607

19. Musilli C, De Siena G, Manni ME, et al. Histamine mediates behavioural and metabolic effects of 3-iodothyroacetic acid, an endogenous end product of thyroid hormone metabolism. *Br J Pharmacol*. 2014;171(14):3476-3484. doi:10.1111/bph.12697

20. Laurino A, Landucci E, Resta F, et al. 3-Iodothyroacetic acid (TA 1), a by-product of thyroid hormone metabolism, reduces the hypnotic effect of ethanol without interacting at GABA-A receptors. *Neurochem Int*. 2018;115:31-36. doi:10.1016/j.neuint.2017.10.008

21. Zucchi R, Rutigliano G, Saponaro F. Novel thyroid hormones. *Endocrine*. 2019;66(1):95-104. doi:10.1007/s12020-019-02018-4

22. Reinwald JR, Weber-Fahr W, Cosa-Linan A, et al. TRIAC Treatment Improves Impaired Brain Network Function and White Matter Loss in Thyroid Hormone Transporter Mct8/Oatp1c1 Deficient Mice. *Int J Mol Sci*. 2022;23(24):15547. doi:10.3390/ijms232415547

23. Piler P, Kandrnal V, Kukla L, et al. Cohort Profile: The European Longitudinal Study of Pregnancy and Childhood (ELSPAC) in the Czech Republic. *Int J Epidemiol*. 2017;46(5):1379+. doi:10.1093/ije/dyw091
